# Molecular signature of progenitor cells isolated from young and adult human hearts

**DOI:** 10.1038/s41598-018-26969-2

**Published:** 2018-06-18

**Authors:** Ann-Sophie Walravens, Maarten Vanhaverbeke, Lara Ottaviani, Hilde Gillijns, Sander Trenson, Nina Vanden Driessche, Aernout Luttun, Bart Meyns, Paul Herijgers, Filip Rega, Ruth Heying, Maurilio Sampaolesi, Stefan Janssens

**Affiliations:** 1 Department of Cardiovascular Sciences, KU Leuven, 3000 Leuven, Belgium; 2 Department of Development and Regeneration, KU Leuven, 3000 Leuven, Belgium

## Abstract

The loss of endogenous cardiac regenerative capacity within the first week of postnatal life has intensified clinical trials to induce cardiac regeneration in the adult mammalian heart using different progenitor cell types. We hypothesized that donor age-related phenotypic and functional characteristics of cardiac progenitor cells (CPC) account for mixed results of cell-based cardiac repair. We compared expression profiles and cell turnover rates of human heart-derived c-kit^pos^ progenitors (c-kit^pos^ CPC) and cardiosphere-derived cells (CDC) from young and adult donor origin and studied their *in vitro* angiogenic and cardiac differentiation potential, which can be relevant for cardiac repair. We report that 3-dimensional CDC expansion recapitulates a conducive environment for growth factor and cytokine release from adult donor cells (aCDC) that optimally supports vascular tube formation and vessel sprouting. Transdifferentiation capacity of c-kit^pos^ CPCs and CDCs towards cardiomyocyte-like cells was modest, however, most notable in young c-kit^pos^ cells and adult CDCs. Progenitors isolated with different methods thus show cell- and donor-specific characteristics that may account for variable contributions in functional myocardial recovery.

## Introduction

The adult mammalian heart is traditionally considered as a terminally differentiated organ, not able to regenerate after massive cell loss. Acute myocardial infarction (AMI), the most severe presentation of ischemic heart disease, remains the leading cause of death worldwide (2013, WHO) and generally implies the loss of approximately 1 billion cardiac myocytes (CM). In survivors of large AMI involving more than 25–30% of left ventricular mass, the heart undergoes a remodelling process with progressive dilatation and functional impairment, resulting in heart failure (HF). The HF syndrome^1^ affects 4% of the population worldwide and carries an ominous prognosis despite state-of-the-art guideline-recommended therapies^2^, emphasizing the need for innovative treatments. To date, the safety and efficacy of multiple candidate cell types, including skeletal myoblasts^3^, heterogeneous bone marrow-derived mononuclear cells^4^ and mesenchymal stem cells (MSC)^5^ have been studied in preclinical and clinical trials of ischemic myocardial damage with mixed results and overall low rates of progenitor cell engraftment and cardiac differentiation. The more recent discovery of endogenous cardiac progenitor cells (CPC) together with the observation of a persistent, yet limited regenerative potential in the adult heart^6^, prompted a shift towards CPCs as a promising candidate for cell-based therapeutic interventions.

In contrast to most tissues, the heart is host to a remarkably extended list of progenitor cells, a discovery that is based on a multiparametric strategy for their phenotypic and functional characterization. The use of different cell surface receptors (c-kit^7^, Sca1^8^), lineage marker cocktails, dye expulsion characteristics typical of side population (SP) phenotype with long-term repopulation capacity (SP cells)^9^ and (non-) adherent growth properties in culture (MSCs^5^, cardiospheres and cardiosphere-derived cells (CDC)^10^ have revealed variable levels of cardiac commitment, not paralleling the capacity for cardiac repair. To what extent this is caused by different transcriptional profiles or by donor age-related functional impairment of heart-derived progenitor cells, remains incompletely understood. The molecular signature of some of these culture expanded heart-derived progenitors has recently been compared in age- and gender-matched mice^11^, but has not yet been established in humans. In this study, we therefore focused on 2 heart-derived progenitor cell populations that have been recently introduced in clinical translation, autologous c-kit^pos^–selected CPCs^12^ and CDCs^13,14^. For the first time we compared molecular signatures and proliferation and differentiation characteristics of progenitor cells from young donor hearts (c-kit^pos^ yCPC and yCDC) with cells obtained from adult donors with advanced ischemic disease (c-kit^pos^ aCPC and aCDC). Here we report that culture expanded CDCs derived from adult donors have a distinct transcriptional profile with greater cell cycle activity and prominent *in vitro* paracrine growth factor release. The molecular signature of aduIt CDCs favours pro-angiogenic, cytotrophic and immunomodulatory effects while cardiac transdifferentiation potential is modest and most notable in c-kit^pos^ yCPCs and aCDCs.

## Results

### Morphological characterization of human adult and young c-kit^pos^ CPCs and CDCs

**Figure 1:**
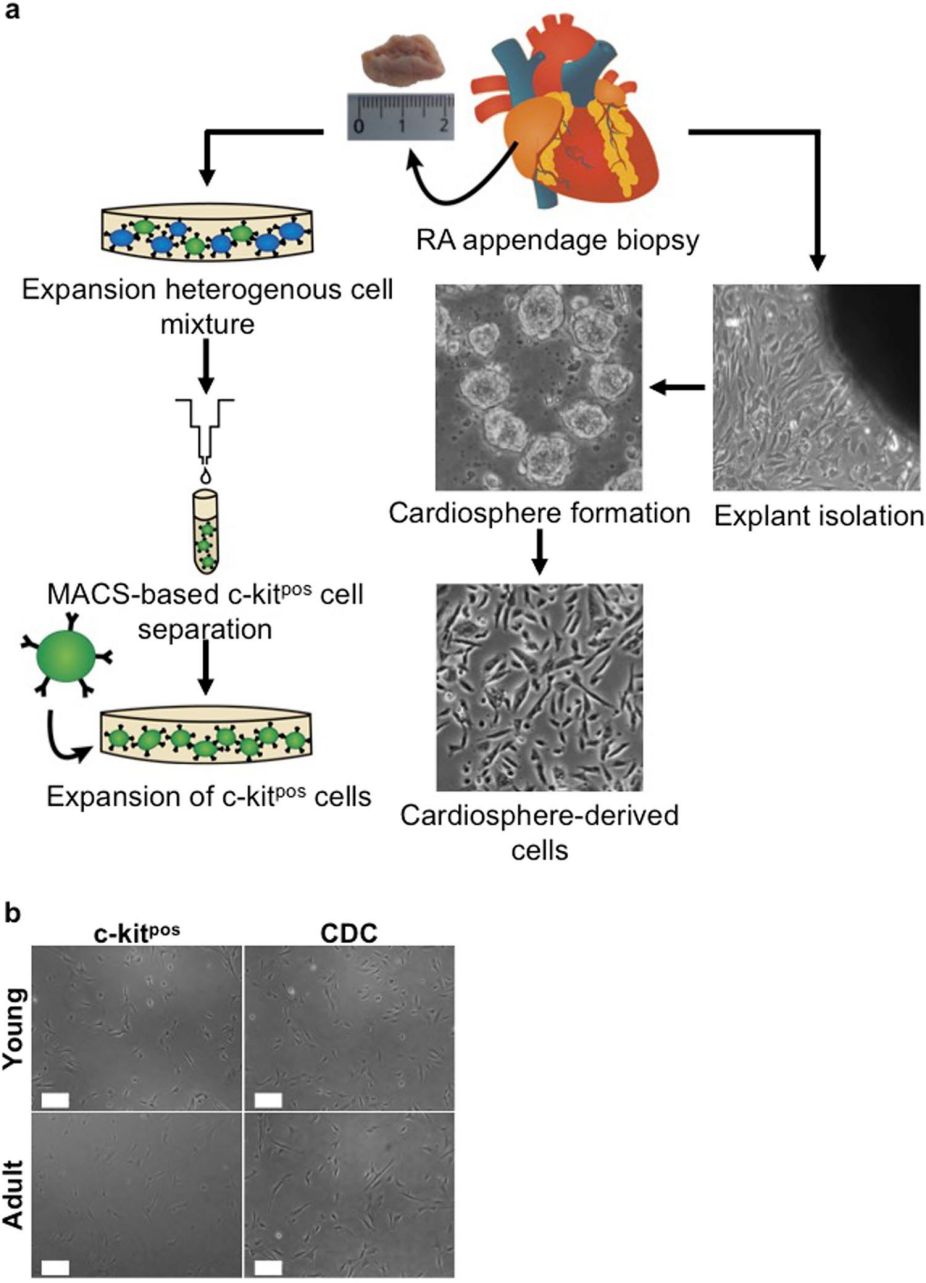
Isolation and morphological comparison of c-kit^pos^ CPCs and CDCs. (**a**) Schematic overview of the steps involved to isolate c-kit^pos^ CPCs and CDCs. To isolate c-kit^pos^ cells, a human RA appendage biopsy is minced into small pieces and collagenase digested to culture a heterogeneous mixture of single cells for 2 passages prior to MACS-based selection. To obtain CDCs, a mild collagenase-based digestion is performed on small myocardial fragments to allow outgrowth of fibroblasts and phase-bright cells from the cardiac explants. The latter are transferred onto a poly-D-Lysine coated dish to form cardiospheres. Finally, the cardiospheres are collected and grown as monolayer cells called CDCs. (**b**) Phase bright-field pictures of c-kit^pos^ CPCs and CDCs derived from young and adult donors. Scale bars represent 200 µm. CPC, cardiac progenitor cell; CDC, cardiosphere-derived cell; RA, right atrial.

To derive progenitor cells from human right atrial (RA) appendage biopsies, two different isolation methods were used (Fig. 1a): positive selection using the surface marker c-kit (CD117) to isolate c-kit^pos^ CPCs and formation of cardiospheres after two weeks of explant culture to derive 2D cultures of CDCs. Phenotypically, CDCs represent a more heterogeneous population of cells irrespective of donor age (young versus adult) compared to c-kit^pos^ cells, but no major differences in shape or size were observed between progenitor cells from young and adult donor hearts at early passages (Fig. 1b) as confirmed with FACS analysis based on FSC plots (data not shown).

### Gene expression profiling of CDCs and c-kit^pos^ CPCs derived from adult and young donors

**Figure 2:**
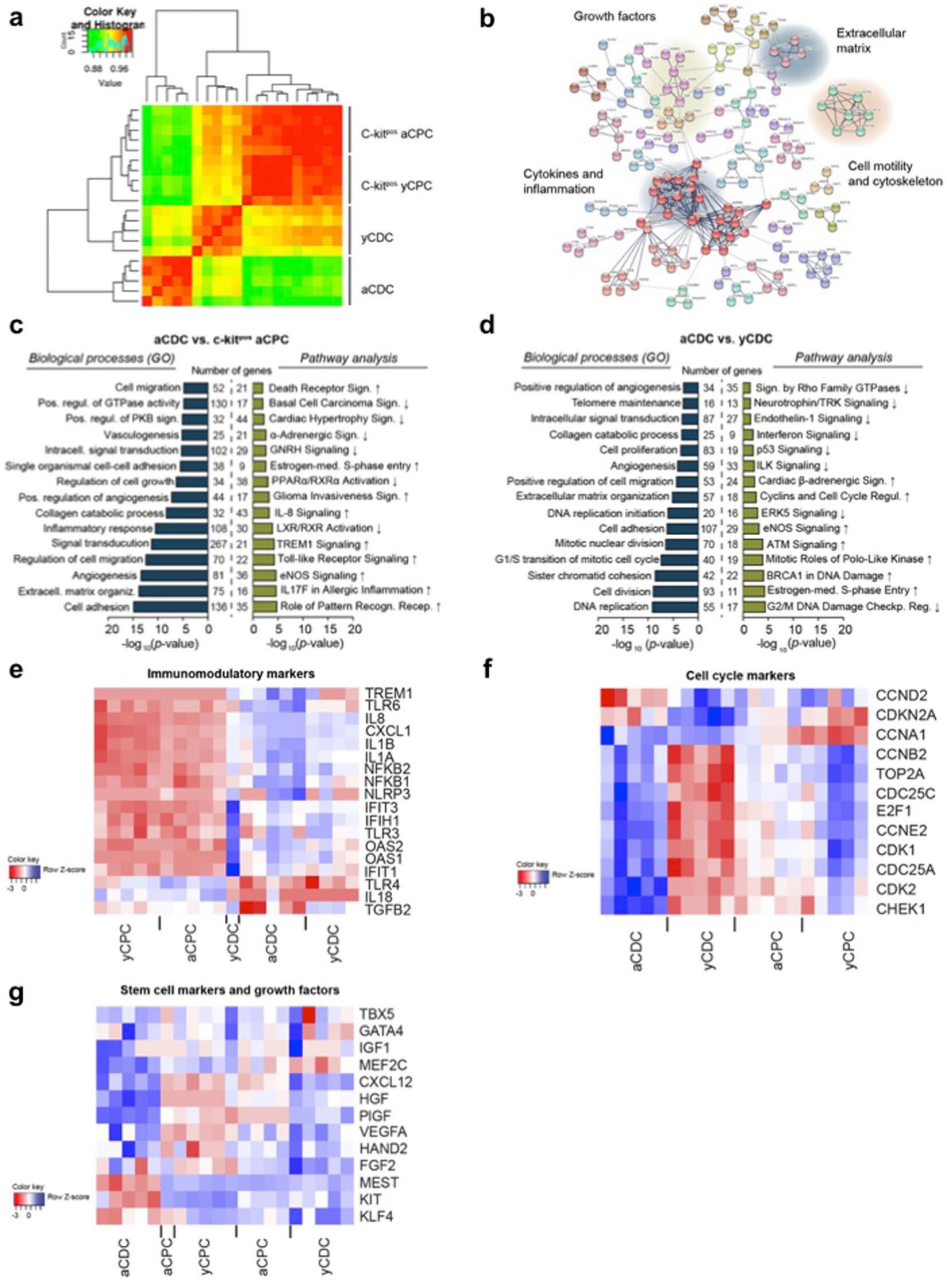
Differential gene expression between CDCs and c-kit^pos^ CPCs derived from adult and young donors. (**a**) Heat map of Spearman correlations with the greatest differences observed between aCDCs and c-kit^pos^ CPCs and between aCDCs and yCDCs. (**b**) String network analysis using the top significant genes showed clusters of transcripts that are related to cytokines and inflammation, growth factors, cell motility and extracellular matrix. (**c**,**d**) Gene ontology results using DAVID software (blue) and pathway analysis (IPA) (green) comparing aCDCs and c-kit^pos^ aCPCs (**c**) and yCDCs and aCDCs (**d**) confirming the string network analysis. Compared to c-kit^pos^ aCPCs, aCDCs are more enriched in inflammatory processes (**c**), while cell cycle pathways are more prominent in aCDCs compared to yCDCs (**d**). (**e–g**) Heatmaps of immunomodulatory genes, cell cycle markers and stem cell markers and growth factors, red = downregulated; blue = upregulated. c-kit^pos^ yCPC (ages 5 d, 2 m, 2.5 m, 3 m, 4 m), c-kit^pos^ aCPC (ages 62 y, 65 y, 74 y, 79 y, 82 y), yCDC (ages 4 d, 1,5 m, 3 m, 5 m, 10 y), aCDC (ages 47 y, 65 y, 70 y, 75 y, 77 y).

To identify biological differences between different progenitor cell populations, we performed RNA sequencing and pathway analysis of CDCs and c-kit^pos^ CPCs derived from adult and young donors. The full list of differentially expressed transcripts and identified processes and pathways is available as an Online Supplement. Based on hierarchical clustering, the greatest contrast was observed between aCDCs and yCDCs and between aCDCs and c-kit^pos^ CPCs derived from young and adult donors (Fig. 2a). In total, 2,886 transcripts showed a significant expression difference of at least 2-fold between aCDCs and yCDCs: 1,423 had higher expression levels and 1,463 had lower expression levels. String network analysis revealed clusters of transcripts related to cytokines and inflammation, growth factors, cell motility and extracellular matrix (Fig. 2b). A second independent approach using gene ontology analysis confirms significant enrichment of biological processes related to the inflammatory response, cell growth, angiogenesis and cell migration in aCDCs compared to c-kit^pos^ aCPCs (Fig. 2c, blue) and enrichment for cell cycle processes, angiogenesis and cell proliferation when comparing aCDCs to yCDCs (Fig. 2d, blue). We then performed pathway analysis and quantified the activation of the identified canonical pathways. More specifically, this revealed significant activation of Toll-like Receptor, TREM1 and IL-8 signalling in aCDCs compared to c-kit^pos^ aCPCs (Fig. 2c, green), and cell cycle related pathways in aCDCs compared to yCDCs (Fig. 2d, green). In addition, eNOS and glioma invasiveness signalling were more activated whereas LXR/RXR and PPARα/RXRα activation, GnRH (Gonadotropin Releasing Hormone) signalling, α-adrenergic, and cardiac hypertrophy signalling were significantly deactivated in aCDCs (Fig. 2c and Table 1).

**Table 1:** Pathway analysis showing the top 5 significantly up- or downregulated pathways of aCDCs versus yCDCs (*P* < 0.05; z-score ≥ |1.5|).

Pathway	z-score	Genes
Cell cycle: G2/M DNA damage checkpoint regulation	−2,324	*TOP2A,BRCA1,AURKA,CDKN2A,CCNB2,CDC25C,BORA,PKMYT1,CKS2,GADD45A,CKS1B,PRKCZ,PLK1,CDK1,CCNB1,CHEK1,CDC25B*
Oestrogen-mediated S-phase entry	2,714	*CCNE2,CCNA1,CCND1,E2F2,CDK2,CDC25A,E2F1,CCNA2,RBL1,ESR1,CDK1*
Role of BRCA1 in DNA damage response	1,500	*BRCA1,RFC3,FANCB,E2F1,BLM,TOPBP1,DPF1,FANCD2,RFC5,FANCE,STAT1,E2F2,SMARCD3,RFC2,FANCG,FANCA,RBL1,GADD45A,BRCA2,RAD51,PLK1,CHEK1*
Mitotic roles of Polo-like kinase	1,807	*KIF23,ESPL1,CDC25A,PRC1,CDC20,CCNB2,KIF11,CDC25C,CDC7,PKMYT1,PLK4,PLK2,PTTG1,HSP90AA1,PLK1,CDK1,CCNB1,FBXO5,CDC25B*
ATM signalling	1,500	*BRCA1,MAPK10,CDK2,CDC25A,GADD45B,CCNB2,SMC2,H2AFX,CDC25C,BLM,FANCD2,CREB5,MAPK13,GADD45A,RAD51,CDK1,CCNB1,CHEK1*

ATM, ataxia telangiectasia mutated; BRCA1, breast cancer 1.

Subsequently, we investigated the identified functional differences on a gene expression level for immunomodulation (Fig. 2e), cell cycle markers (Fig. 2f) and stem cell markers and growth factors (Fig. 2g). With respect to immunomodulation, many inflammatory genes including *IL1B*, *IL8* (*CXCL8*) and *TGFB1* were higher expressed in aCDCs compared to yCDCs, but also when compared to c-kit^pos^ aCPCs (Fig. 2e). The difference between CDCs and c-kit-selected CPCs is predominantly driven by greater inflammatory signalling (Toll-like receptors *TLR*, *TREM1*, *IL8*) with the highest activation in aCDCs, an intermediate profile in yCDCs, and the lowest expression levels in c-kit^pos^ CPCs irrelevant of donor age. Other transcripts including *IL18*, *TGFB2* and *TLR4*, however, are lower expressed in aCDCs compared to yCDCs. The expression profile of cell cycle-related transcripts in aCDCs is consistent with greater proliferation potential when compared to yCDCs, while this difference appears to be less pronounced in c-kit-selected CPCs with a greater proliferation potential for the c-kit^pos^ yCPCs (Fig. 2f). For the transcription factors, only *MEF2C* was higher expressed in aCDCs compared to yCDCs and c-kit^pos^ CPCs isolated from adult and young donors. In contrast, the expression of *MEST*, a member of the hydrolase superfamily important in early mesoderm formation, is the lowest in aCDCs while *TBX5* and *GATA4* had comparable transcript levels in all progenitor cell populations. The expression of angiogenic growth factors *CXCL12* (stromal-derived factor-1 alpha (SDF1-α)), *HGF* (hepatocyte growth factor), *PlGF* (placental growth factor), *VEGFA* (vascular endothelial growth factor A) and *FGF2* (fibroblast growth factor 2, or basic fibroblast growth factor) is more highly expressed in aCDCs and to a lesser extent in yCDCs compared to c-kit^pos^ aCPCs and c-kit^pos^ yCPCs. Finally, the stem cell factors *KIT* and *KLF4* are the lowest in aCDCs compared to yCDCs and c-kit^pos^ CPCs derived from young and adult donors (Fig. 2g).

Taken together and when compared to c-kit^pos^ aCPCs, aCDCs display a higher activation of cell cycle pathways, inflammatory pathways important for reparative responses (TLR, TREM1, Pattern Recognition Receptors), critical growth factors and eNOS signalling, all of which may contribute to a unique cardioprotective capacity during cytotoxic stress conditions.

### Gene signature and proliferation capacity of heart-derived c-kit^pos^ CPCs and CDCs

**Figure 3:**
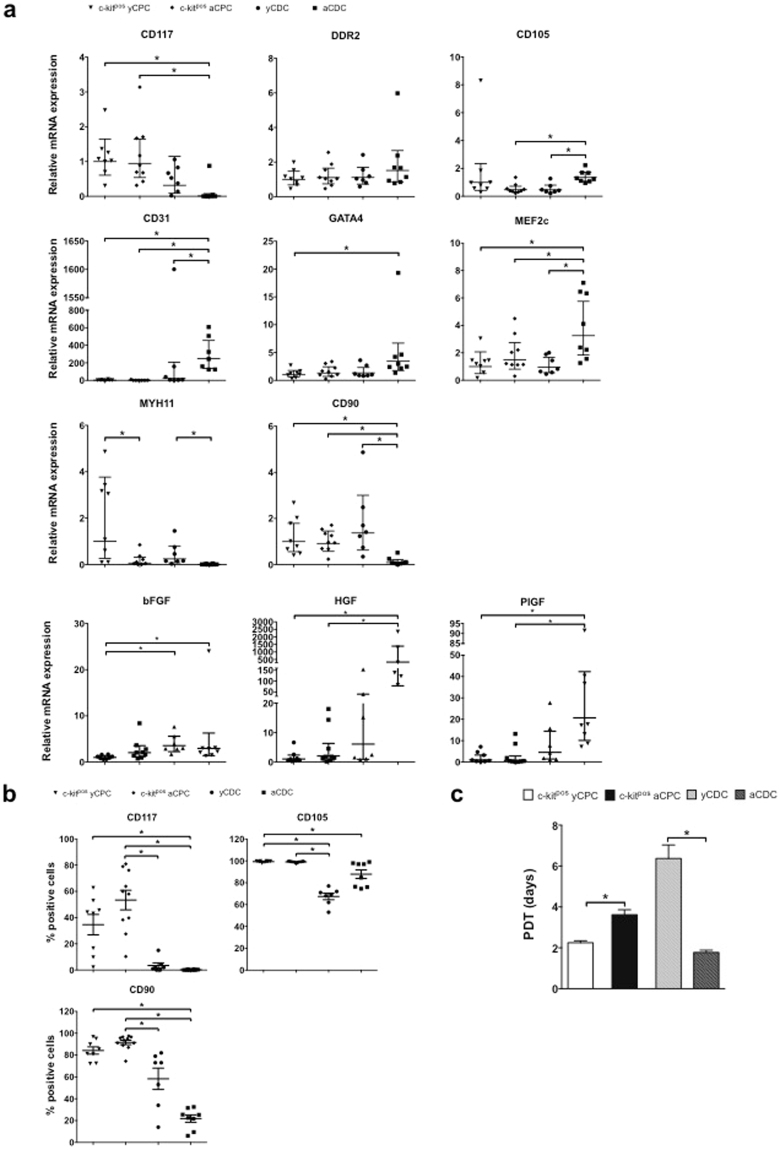
Phenotypic comparison of adult and young c-kit^pos^ CPCs and CDCs. (**a**) Gene expression levels of *CD117* (*KIT*), *CD105*, *CD90*, the EC marker *CD31*, early cardiac transcription factors *GATA4* and *MEF2c*, SMC marker *MYH11* and fibroblast marker *DDR2*. c-kit^pos^ yCPC (n = 8), c-kit^pos^ aCPC (n = 9), yCDC (n = 7) and aCDC (n = 7). Results are geometric mean with 95% CI, one-way ANOVA was performed for normally distributed data and Kruskal-Wallis test for non-normally distributed data, *p < 0.05. EC, endothelial cell; SMC, smooth muscle cell; CPC, cardiac progenitor cell; CDC, cardiosphere-derived cell. (**b**) Flow cytometry analysis of c-kit^pos^ yCPCs (n = 8), c-kit^pos^ aCPCs (n = 10), yCDCs (n = 7) and aCDCs (n = 8) for CD90, CD105, and CD117. All analyses were performed after 6 passages. Results are mean ± SEM, *p < 0.05. (**c**) PDT of c-kit^pos^ yCPCs (n = 4), c-kit^pos^ aCPCs (n = 5), yCDCs (n = 5) and aCDCs (n = 4). Results are mean ± SEM, *p *<* 0.05. CPC, cardiac progenitor cell; CDC, cardiosphere-derived cell; PDT, population doubling time.

To confirm RNA sequencing expression data, we performed qPCR analysis and measured significantly higher *CD117* (c-kit) transcript levels in c-kit^pos^ yCPCs and c-kit^pos^ aCPCs compared to aCDCs (Fig. 3a) and within the CDC population no significant difference was found in *CD117* levels. The fibroblast marker *DDR2*, was equally expressed in young and adult c-kit^pos^ CPCs and CDCs. The expression of the proliferation-associated and hypoxia-inducible endoglin *CD105* was prominent in all four cell populations but significantly higher in aCDCs compared to yCDCs and c-kit^pos^ aCPCs (p < 0.05). Conversely, aCDCs showed significantly lower gene expression level of the mesenchymal marker *CD90* compared to yCDCs and c-kit^pos^ yCPCs and c-kit^pos^ aCPCs (p < 0.05). To study lineage commitment of human progenitor cells, expression levels of the endothelial cell (EC) marker *CD31*, the early cardiac transcription factors *MEF2c* and *GATA4* and the smooth muscle cell (SMC) marker *MYH11*, were measured. Interestingly, *CD31* and *MEF2c* expression was significantly higher in aCDCs compared to yCDCs and c-kit^pos^ CPCs derived from young and adult donors (p < 0.05). The same trend was observed for *GATA4* expression however the expression was only significant compared to c-kit^pos^ yCPC. The expression level of the SMC marker *MYH11* was significantly higher in cells derived from young donors compared to cells derived from adult donors irrespective of the isolation method (p < 0.05).

Because heart-derived progenitor cells have important paracrine effector mechanisms, we compared gene expression of different candidate growth factors and cytokines. Transcript levels of *FGF2* were significantly higher in yCDCs and aCDCs than in c-kit^pos^ yCPCs (p < 0.05). In addition, the expression of *HGF* and *PlGF*, was significantly higher in aCDCs compared to c-kit^pos^ yCPCs and c-kit^pos^ aCPCs (p < 0.05 and p < 0.01, respectively). Finally, *IGF-1* and insulin growth factor-receptor2 (*IGF-2R*) gene expression were significantly higher in aCDCs than in c-kit^pos^ aCPCs (p < 0.05; Fig. S1). Also, the expression of inflammation markers *IL1B*, *IL8* and *TGFB1* were higher in aCDCs compared to yCDCs, which confirms the RNA sequencing data (Fig. S2). In addition, *TGFB1* expression was highest in aCDCs compared to yCDCs, c-kit^pos^ yCPCs and c-kit^pos^ aCPCs (data not shown).

The presence of the selection marker c-kit after 5 to 6 passages in culture was also verified using flow cytometry analysis (Fig. 3b). Consistent with quantitative gene expression data, c-kit^pos^ CPCs from adult and young donors expressed CD117 (KIT) to variable degrees (53 ± 7% and 35 ± 8%, respectively). In contrast, CD117 was almost undetectable in aCDCs (0.2 ± 0.1%) and very low in yCDCs (3.4 ± 2.0%). Cell surface expression of the mesenchymal marker CD90 (THY-1) was significantly higher in c-kit^pos^ CPCs (aCPC, 91 ± 2%; yCPC, 84 ± 3%) than in aCDCs (22 ± 3%, p < 0.05) and in addition significantly higher in c-kit^pos^ aCPC compared to yCDCs (58 ± 10%). This protein plays an important role in cell-cell and cell-matrix interactions and expression levels have been reported to be inversely related to regenerative capacity of CPCs^15^. CD105 protein, an accessory TGF-beta receptor involved in angiogenic signalling, was expressed on the surface of virtually all progenitors but to a lesser extent on CDCs (c-kit^pos^ yCPC, 99 ± 0.3%; c-kit^pos^ aCPC, 99 ± 0.2%; yCDC, 67 ± 3.0%; aCDC, 88 ± 4%; yCDC compared to c-kit^pos^ CPCs p < 0.001; aCDC versus c-kit^pos^ yCPC p < 0.05).

To evaluate proliferation capacity of the different progenitor cell types, we calculated population-doubling times (PDT) (Fig. 3c). Consistent with observed differences in transcript levels of cell-cycle regulated genes (Fig. 2f), c-kit^pos^ yCPCs have a significantly lower PDT and thus higher proliferation rate compared to c-kit^pos^ aCPCs (2.25 ± 0.10 days versus 3.62 ± 0.24 days, p < 0.02). In contrast, adult progenitor cells isolated according to the explant culture technique (aCDC) showed significantly lower PDTs and thus higher proliferation rates than yCDCs (1.78 ± 0.11 days versus 6.37 ± 0.66 days, p < 0.02). In addition, the average passage number at which proliferation of the cells stagnated or started to decline, was significantly lower in c-kit^pos^ aCPCs and yCDCs (13.0 ± 0.7 and 12.1 ± 0.7 passages, respectively), the cell groups with the highest PDTs, compared to c-kit^pos^ yCPCs and aCDCs (21.3 ± 3.0 and 22.0 ± 0.4 passages, respectively).

### Vasculogenic and angiogenic potential of c-kit^pos^ CPCs and CDCs derived from adult and young donors

**Figure 4:**
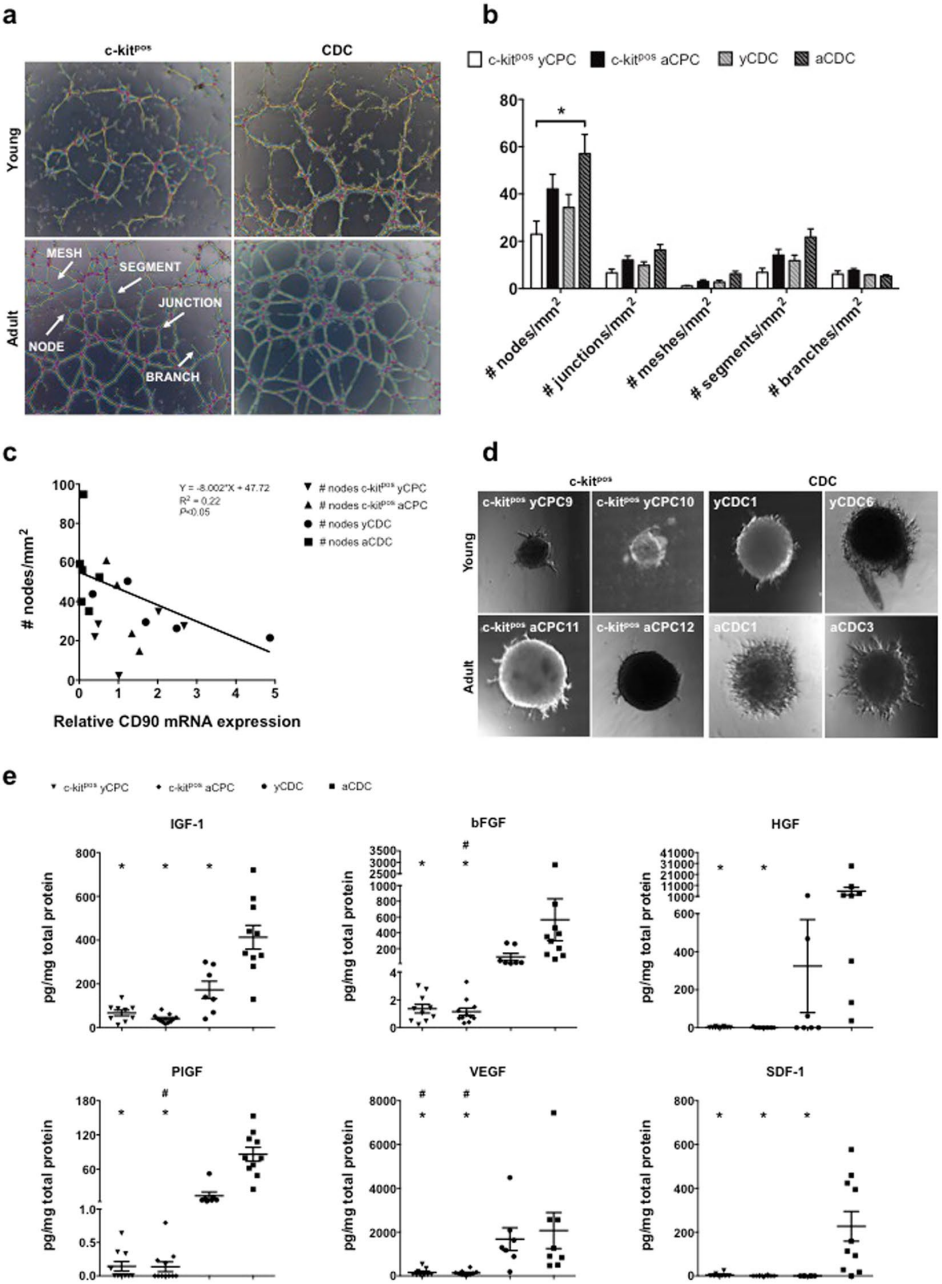
Vasculogenic and angiogenic potential of adult and young c-kit^pos^ CPCs and CDCs. (**a**) Analysis of network formation 6 hours after culture on Matrigel® of c-kit^pos^ yCPCs (n = 5), c-kit^pos^ aCPCs (n = 8), yCDCs (n = 5) and aCDCs (n = 9). (**b**) Quantification of number of nodes, junctions, meshes, segments and branches/mm^2^. Results are mean ± SEM, *p < 0.05. (**c**) Correlation of vasculogenic potential, expressed as number of nodes/mm^2^ and *CD90* mRNA expression of aCDCs and yCDCs. (**d**) Representative images of 3D sprouting of c-kit^pos^ yCPC (n = 2), c-kit^pos^ aCPC (n = 2), yCDC (n = 2) and aCDC (n = 2) spheroids after 48 hours incubation. Per group, cells of 2 different donors were used and for each donor, 4 different spheroids were imaged. One representative spheroid per donor is depicted. (**e**) Growth factor release analysis in conditioned medium collected after 24 hours incubation of c-kit^pos^ yCPCs (n = 9), c-kit^pos^ aCPCs (n = 11), yCDCs (n = 7) and aCDCs (n = 10) for IGF-1, VEGF, bFGF, PlGF, HGF and SDF-1. Results are mean ± SEM, *p < 0.05 compared to aCDC and ^#^p < 0.05 compared to yCDC. CPC, cardiac progenitor cell; CDC, cardiosphere-derived cell.

Next, the vasculogenic potential of human progenitors was evaluated after 6 hours culture of the cells on Matrigel® (Fig. 4a–c). To develop firmly established vascular networks, progenitor cells must be able to sprout, form branches and interconnect them into segments, bounded by a junction or node (Fig. 4a). The number of branches was similar between the 4 different cell populations (c-kit^pos^ yCPC, 6.1 ± 1.4/mm^2^; c-kit^pos^ aCPC, 7.6 ± 0.8/mm^2^; yCDC, 5.76 ± 0.17/mm^2^; aCDC, 5.2 ± 0.5/mm^2^). However, the ability to form vascular networks, as reflected by the number of nodes, was significantly lower for c-kit^pos^ yCPCs (c-kit^pos^ yCPC, 23.0 ± 5.6/mm^2^) compared to progenitor cells isolated from adult donors (aCDC, 57.1 ± 8.1/mm^2^). Of note, yCDCs show a trend for a higher tube formation potential when compared to c-kit^pos^ yCPCs (Fig. 4a,b). These relative differences in vascular network formation may in part be attributable to variable *CD90* mRNA and protein expression patterns (Fig. 3), which have been shown in previous studies to inversely correlate with endothelial transdifferentiation and regeneration capacity^15^. The CD90 expression in yCDCs varied between the different donors (Fig. 3). To investigate if variation in CD90 expression is associated with differences in tube formation capacity, we measured the correlation between *CD90* mRNA expression and the number of nodes. Here, we report an inverse correlation between the number of nodes and *CD90* mRNA expression of aCDCs and yCDCs (Fig. 4c). Because the *in vitro* Matrigel® assay does not allow evaluation of vascular sprouting and formation of lumen structures, a fundamental property of ECs, we complemented our analysis with an *in vitro* 3D spheroid assay in Matrigel® to study sprouting angiogenesis (Fig. 4d). The additional 3D spheroid assay confirmed the superior potential of aCDCs (n = 2) to sprout and form a vascular network compared to yCDCs (n = 2), c-kit^pos^ yCPCs (n = 2) and c-kit^pos^ aCPCs (n = 2). Taken together, our data indicate that all human progenitor types have an intrinsic vasculogenic potential, however, progenitors isolated from young donors are possibly more immature, with reduced endothelial lineage commitment resulting in less developed vascular networks.

To further explore the angiogenic phenotype, growth factor secretome was analysed in medium conditioned by human CPCs using ELISA for IGF-1, bFGF, HGF, PlGF, VEGF and SDF-1 (Fig. 4e). Consistent with qRT-PCR data, the secretion of IGF-1, HGF, bFGF, VEGF and PlGF was significantly higher in CDCs compared to c-kit^pos^ CPCs, irrespective of donor age (*p < 0.05 versus aCDC; ^#^p < 0.05 versus yCDC). In addition, within the CDC population the secretion of IGF-1 was significantly lower in cells derived from young hearts. Of note, the secretion of the chemotactic protein SDF-1, a key regulator of stem cell migration to sites of tissue injury^16^, was undetectable in c-kit^pos^ CPCs derived from adult and young donors and in yCDCs but prominent in the secretome obtained from aCDCs (p < 0.01).

Finally, because of the recently recognized important role of exosomes, small extracellular membrane vesicles containing proteins, mRNA and miRNA that are secreted by cells to induce cellular communication, we measured exosome levels in 48 hours serum-free conditioned medium. Nanoparticle analysis technology to determine the concentration and particle distribution demonstrated that exosomes derived from aCDCs and yCDCs represented a more homogeneous and purified population than those shed by c-kit^pos^ aCPCs and yCPCs and contain variable concentrations of defined miRNAs with a presumed cyto- or cardioprotective effect^17^ (Fig. S3A,B).

### Cardiac differentiation potential of c-kit^pos^ CPCs and CDCs derived from adult and young donors

**Figure 5:**
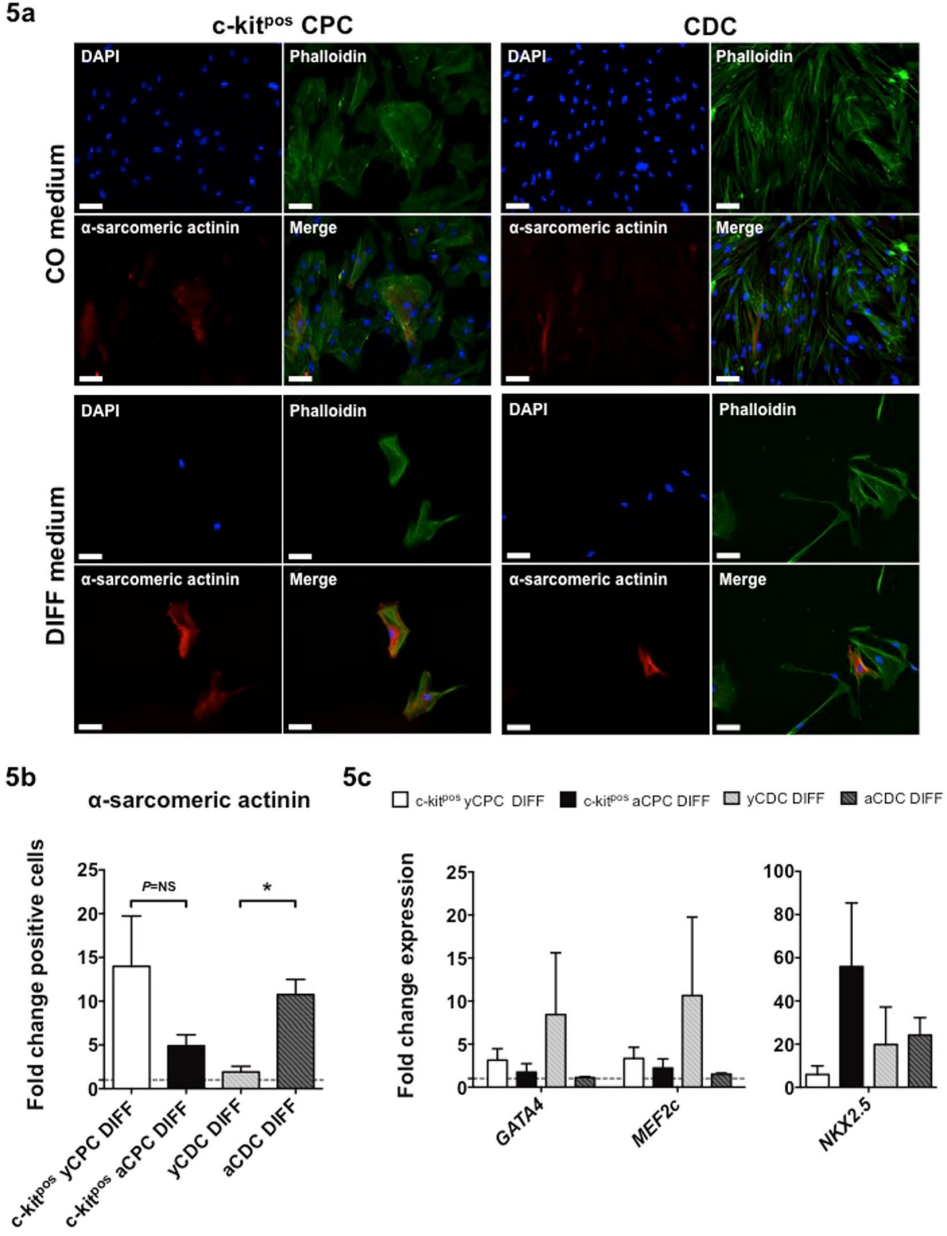
The cardiac differentiation capacity of adult and young c-kit^pos^ CPCs and CDCs. (**a**) Double immunofluorescence staining of phalloidin (green) and α-sarcomeric actinin (red) revealed protein expression of a cardiac contractile protein after incubation in cardiac differentiation medium (DIFF) or regular expansion medium (CON) for 12 days. c-kit^pos^ yCPCs (n = 5), c-kit^pos^ aCPCs (n = 6), yCDCs (n = 5) and aCDCs (n = 5). Scale bars represent 100 µm. (**b**) Quantification of the immunofluorescent staining for α-sarcomeric actinin positive cells after 12 days incubation in DIFF medium. Fold change of alpha-sarcomeric actinin positive cells in DIFF treated groups compared to CON groups, results are mean ± SEM, *p < 0.05. (**c**) Fold change expression of *GATA4*, *MEF2c* and *NKX2.5* in DIFF treated groups compared to CON groups. Results are mean ± SEM.

To test the hypothesis that heart-derived progenitors from young donors have a greater cardiac differentiation potential than progenitors derived from adult donors, all cell populations were incubated with cardiac differentiation medium (DIFF) or regular expansion medium (CON). Immunofluorescence stainings showed the presence of the cardiac contractile protein α-sarcomeric actinin in CDCs and c-kit^pos^ CPCs after 12 days incubation with DIFF medium (Fig. 5a,b). Contrary to our hypothesis, we observed a greater increase of α-sarcomeric actinin-positive cells (DIFF compared to CON) under these culture conditions in aCDCs compared to yCDCs. However, the opposite effect was observed in ckit^pos^ CPCs, with a greater increase of α-sarcomeric actinin-positive cells in c-kit^pos^ yCPCs compared to c-kit^pos^ aCPCs (Fig. 5b). This suggests a greater cardiac differentiation potential of c-kit^pos^ yCPCs compared to c-kit^pos^ aCPCs, yCDCs and aCDCs. Of note, no contractile phenotype was observed after 12 days incubation with DIFF medium. In addition, expression of the cardiac lineage markers *GATA4*, *MEF2c* and *NKX2.5* confirmed activation of the cardiac differentiation program in all cells treated with cardiac differentiation medium (DIFF) (Fig. 5c), which is consistent with the increased α-sarcomeric actinin-positive cells following incubation in DIFF medium (Fig. 5a,b).

### Culture expansion conditions do not explain the observed differences in cell phenotype and functional activity between CDCs and c-kit^pos^ CPCs

Finally, the cell phenotype and functional activity was investigated after culturing CDCs (yCDC, n = 2; aCDC, n = 2) in c-kit^pos^ CPC expansion medium and c-kit^pos^ CPCs (c-kit^pos^ yCPC, n = 2; c-kit^pos^ aCPC, n = 2) in CDC expansion medium on fibronectin-coated culture plates for at least 2 passages. The expression levels of *CD117*, *CD105* and *CD90* in these cells cultured in their counterpart medium (SWAP) was tested with qPCR and compared to the original transcriptome as reported in Fig. 3a (Fig. S4A). We measured similar expression profiles for all 3 genes irrespective of the expansion medium with the highest expression of *CD117* in c-kit^pos^ yCPCs. *CD105* was equally expressed in all cell groups and *CD90* showed again the lowest expression in aCDCs. Next, the vasculogenic potential of the four cell groups was explored after culturing the progenitors with their counterpart medium (SWAP). Again, results showed that the ability to form vascular networks, as reflected by the number of nodes, was lower in progenitors isolated from young donors compared to adult donors (Fig. S4B). In addition and again in concert with our original observations (Fig. 4a,b), the number of junctions, meshes, segments and branches showed a comparable pattern with progenitors derived from adult donors forming more complex networks compared to progenitors derived from young donors. In conclusion, these data suggest that CDC and c-kit^pos^ CPC culture conditions do not account for the phenotypic and functional differences between the four progenitor groups.

## Discussion

Recent evidence indicates that the mammalian heart harbours multiple cardiogenic progenitor cell populations and stages a marked endogenous regenerative response after tissue damage in early postnatal life^18^. Because this capacity for scar-free healing through cardiomyogenesis is lost in the adult heart, intracardiac transfer of c-kit^pos^ CPCs and a heterogeneous explant population of CDCs has been introduced in clinical trials in an effort to enhance cardiac repair after ischemic injury. In the present study, we aimed to bridge the gap in our understanding of the molecular signature of these 2 human heart-derived progenitor cell populations obtained at different stages in postnatal life. We compared *in vitro* the phenotypic, functional and genetic characteristics of heart-derived progenitor cells from neonates and young patients with congenital heart disease undergoing corrective surgery and from adult patients with advanced ischemic and valvular disease. We deliberately studied culture expanded cells after 4 to 5 passages as was peformed in pilot clinical studies (e.g. SCIPIO^12^ and CADUCEUS^13,14^ trials) and observed a distinct molecular signature between CDCs of different donor age, but not between c-kit-selected progenitor cells of young and adult patients. The molecular pathways with different states of activation or repression between adult and young CDCs play a pivotal role in cell survival, proliferation and endothelial cell (EC) or cardiac myocyte-specific functions. Importantly, adult heart-derived and culture expanded CDCs showed significantly higher activation of the EC-associated *NOS3* gene, showed less *CD90* surface gene expression and secreted high levels of pro-angiogenic cytokines and growth factors. These phenotypic characteristics conferred greater capacity for vascular tube formation and sprouting in a 3D spheroid assay. Transcriptional profiling also markedly contrasted aCDCs with c-kit^pos^ CPCs from young and adult heart as evidenced by the consistent activation of cell cycle regulatory genes and of interest, a robust induction of immunomodulatory genes.

Transcriptome analysis of aCDCs revealed a baseline upregulation of myocyte-specific transcription factors (*GATA4* and *MEF2c*) and the EC-specific marker (*CD31*), a constituent of the endothelial intercellular junction, also designated as *PECAM-1* (platelet endothelial cell adhesion molecule-1). Adult CDCs also expressed significantly higher levels of endoglin or *CD105*, known to play a crucial role in angiogenesis^19^, but contrary to MSCs^20^ co-expression of *CD90* was significantly lower. *CD90* expression is lower in aCDCs compared to yCDCs, c-kit^pos^ yCPCs and c-kit^pos^ aCPCs. In fact, it has previously been shown that injection of CDCs expressing high levels of CD90, is associated with a reduced reparative capacity and in contrast CD90^neg^ CDCs induce a more pronounced therapeutic effect^15^. Of interest, this inverse association is also clearly evidenced in our *in vitro* network formation assay where aCDCs induce markedly complex vascular network development. Taken together, transcriptional analysis suggests that heterogeneous aCDCs express early lineage-specific genes, some of which recapitulate an *in vivo* pattern in newly formed cells derived from activation and commitment of resident progenitor cells after injury^21^.

Somewhat unexpectedly, aCDCs had also a significantly lower PDT and thus faster cell turnover compared to their counterparts from young hearts, but this observation was consistent with the significantly higher expression of cyclins and cell cycle regulatory genes. The faster cell turnover in aCDCs than in yCDCs was not recapitulated in c-kit^pos^ CPCs, where cells from adult donors showed a significantly slower cell turnover than c-kit^pos^ yCPCs. A recent study showed that human Isl1^pos^ c-kit^pos^ CPC isolated from adult patients harbor an altered miRNA expression profile that correlated with a reduced proliferation capacity and abrogated response to growth factors compared to Isl1^pos^ c-kit^pos^ CPC derived from neonates^22^. Another study, comparing CDCs derived from patients of various ages, showed limited differences in proliferation capacity, angiogenic potential and expression of growth factors between CDCs from young and elderly patients (≥65 years)^23^. However, in this study the youngest donor was 2 years old, the median age 72.5 years and 61.5% of them were 65 years or older which is in striking contrast with our study. The CDCs derived from young patients were probably underrepresented compared to the CDCs derived from elderly patients to see significant differences whereas in our study CDCs from much younger donors were studied. In contrast, S. Kaushal and co-workers compared CDCs and c-kit^pos^ CPCs derived from donors with various ages, and reported a greater *in vivo* regenerative capacity and a higher secretion of angiogenic factors in CDCs derived from neonates (<30 days old)^24,25^. In our study, we only have 1 and 2 neonates included in yCDC and c-kit^pos^ yCPC, respectively, and this underrepresentation might account for the different results. Also, CDCs from different young ages (neonates, infants and children) showed a comparable *in vivo* regenerative potential^26^. However, in contrast to our study, the angiogenic potential was not investigated in this study. Several studies showed that cell-based therapy improves cardiac function through paracrine mechanisms (mediation of angiogenesis, anti-apoptosis and recruitment of endogenous progenitors) since incorporation of injected cells is rare event^10,27^. In this study, aCDCs show a broad expression panel of angiogenic cytokines and potent cytoprotective growth factors, including IGF-1, HGF, bFGF, PlGF, VEGF, and SDF-1, all of which may mediate the paracrine effects of CDCs. Also, heart-derived CDCs displayed a strikingly distinct exosome release pattern when compared to CPCs. These exosomes gained a lot of interest in view of their important role in paracrine and autocrine signalling^28^. In addition, injection of cardiac progenitor-derived exosomes in an AMI rat model protected CM against apoptosis, showed enhanced angiogenesis and improved cardiac function^29^. However, follow-up studies need to examine to what extent exosome-mediated signalling can affect molecular and cellular pathways that are critically important for cardiac repair or protection. Also, a direct correlation of a particular secreted factor(s), the importance of exosomes as a mediator and *in vitro* tube formation was not performed in this current study.

Finally, we investigated the cardiogenic potential of CDCs and c-kit^pos^ CPCs in an *in vitro* differentiation assay. C-kit^pos^ CPCs derived from young donors and aCDCs showed a tendency for greater transdifferentiation capacity into cardiomyocyte-like cells than c-kit^pos^ aCPCs and yCDCs. The greater commitment towards cardiac lineages for c-kit^pos^ yCPCs and aCDCs and the marked immunomodulatory signature and neovascularization potential of aCDCs represent interesting characteristics for future cell transfer strategies. In chronic ischemic cardiomyopathy, the maladaptive immune activation that accompanies ischemic dysfunction would require a combined intervention that at the same time mitigates excessive immune activation while supporting neovascularization and cardiomyogenesis. Recent reports have called our attention for immunomodulatory properties of cardiac progenitors that are capable of generating an antioxidant, anti-fibrotic, and anti-inflammatory environment^30^. Additionally, de Couto *et al*. showed that injection of CDCs or CDC exosomes in a rat AMI model reduces the number of pro-inflammatory macrophages and simultaneously polarizes the macrophages into a cardioprotective phenotype resulting in less CM apoptosis and reduced infarct size^31,32^. We observed greater activation of some inflammatory transcripts in aCDCs compared to yCDCs and c-kit^pos^ CPCs, while other transcripts including TLR4, implicated in post-infarction inflammation^33^, were less expressed. Pro-inflammatory cytokines IL1B and IL8 are both more highly expressed in aCDCs and promote inflammatory leukocyte recruitment and activation, while IL1 delays myofibroblast activation and the potent pro-angiogenic IL8 contributes to infarct neovascularization^34^. TGF-β is a key player in the transdifferentiation of fibroblasts into myofibroblasts which secrete ECM proteins to form fibrotic scar tissue^35^. We measured higher TGFB1 expression in aCDCs compared to yCDCs and c-kit^pos^ CPCs, but lower TGFB2 levels. IL18 stimulates IFN-γ secretion by NK-cells and T-cells which leads to stimulation of pro-inflammatory macrophages^36^. Our data show that IL18 transcripts are lower in aCDCs compared to yCDCs and c-kit^pos^ CPCs. Taken together, the biological significance of these variable inflammatory gene expression patterns remains incompletely understood and requires targeted *in vivo* studies in the context of cardiac dysfunction.

Several limitations need to be considered in our study. First, the limited amount of RNA available from human progenitor cells precludes transcriptional analysis of a wider panel of genes using qRT-PCR. Second, size constraints of myocardial biopsies precluded cell biology studies on freshly isolated and non-passaged cells and prompted standardized analysis on multi-passaged cells with similar expansion profiles and passage numbers as used in clinical trials. At the same time, limited tissue availability did not allow parallel isolation protocols for both cell types from identical tissue samples. Although *ex vivo* culture expansion may affect genetic signatures, the consistency of the data reported in our analysis irrespective of expansion medium likely reflects the imprinted phenotype of the cells as used in recent clinical trials. Third, the great age range present in the c-kit^pos^ yCPC and yCDC groups and the underrepresentation of neonates may mask the potential of c-kit^pos^ CPCs and CDCs derived from neonates, which should be addressed in further studies. Fourth, we acknowledge the importance of unraveling the factors responsible for the functional effects in our *in vitro* studies, which needs to be addressed in follow-up studies. Finally, additional mechanisms operate *in vivo* including stimulation of resident cardiac cells and cytoprotective effects on ischemic cells, which have not been tested directly in this *in vitro* study.

In conclusion, phenotypic analysis of aCDCs reveals paracrine and immunomodulatory effects that appear to be distinct from those in yCDCs. In addition, c-kit^pos^ yCPCs and aCDCs comprise a greater differentiation potential into cardiomyocyte-like cells. Whether these *in vitro* cell-specific findings correlate with clinically significant changes and whether combination of different progenitor cell types may confer greater clinical benefit than either cell type alone, needs to be addressed in targeted studies.

## Methods

### Tissue samples

The tissue procurement protocol was approved by the Medical Ethics Committee of the University Hospitals Leuven (study number S56078) and all experiments were performed in accordance with the Medical Ethics Committee guidelines. Additionally, informed consent was provided by all adult patients (47–84 years old; c-kit^pos^ aCPC, *n* = 12; aCDC, *n* = 9) and by legal guardians or parents for all young patients (1 day – 14 years old; c-kit^pos^ yCPC, *n* = 10; yCDC, *n* = 7). Main patient characteristics are shown in Tables 2 and S3.

**Table 2:** Clinical characteristics of the patients.

	aCPC c-kit^pos^	yCPC c-kit^pos^	aCDC	yCDC
Number of biopsies	17	15	9	7
Age range	53–84 year		47–77 year	
Days		2		1
1.5–12 months		11		5
>1 year		2		1
Gender (% male)	53	27	89	71
**Cardiovascular risk factors**				
Hypertension (%)	92	NA	56	NA
Diabetes (%)	25	NA	0	NA
Hyperlipidaemia (%)	91	NA	67	NA
Smoking (%)	50	NA	33	NA
**Aetiology (** ***n*** **)**				
Tetralogy of Fallot	NA	1	NA	4
ASD and/or VSD	NA	6	NA	2
TGA	NA	1	NA	1
MVP/MVR ± CABG	4	NA	4	NA
AVP/AVR ± CABG	3	NA	2	NA
Complex valvular disease	4	NA	3	NA
Other	1	2	0	0

CPC, cardiac progenitor cell; CDC, cardiosphere-derived cell; ASD, atrial septal defect; VSD, ventricular septal defect; TGA, transposition of the great arteries; MVP/R, mitral valve repair/replacement; AVP/R, aortic valve repair/replacement; CABG, coronary artery bypass grafting; NA, not applicable.

### Isolation and culture of human progenitor cells

Cardiac tissue samples were obtained from the RA appendage that was routinely discarded from patients undergoing intra-cardiac surgeries. Patients undergoing extra-cardiac surgery were excluded. All adult patients were referred for valve surgery, assist device implantation or heart transplantation. Neonatal RA appendage specimens were collected during corrective surgery for congenital heart disease. No other specific inclusion criteria were used. Human c-kit^pos^ (CD117) CPCs were obtained after collagenase-based digestion of a RA appendage tissue sample based on the methods used in the SCIPIO trial^12^. Briefly, adipose tissue was removed and tissue samples were minced into small pieces before collagenase-based (Collagenase NB4 Standard Grade from Clostridium histolyticum, Serva Electrophoresis) digestion in Ham’s F12 medium containing 1–3 mg/mL collagenase. The supernatant containing single cells were collected and plated in Ham’s F12 medium (Lonza) supplemented with 10% FBS (ThermoFisher Scientific), 1% penicillin/streptomycin (5000 units/mL penicillin, 5000 µg/mL streptomycin; ThermoFisher Scientific), 5 mU/mL human erythropoietin (EPO) (Sigma-Aldrich). When 80–90% confluency was reached, positive selection for c-kit by magnetic-activated cell sorting (MACS) (CD117 microbead kit, Miltenyi Biotec) was performed. Medium was changed twice a week and cells were passaged when 80–90% confluency was reached. Human CDCs were derived according to the protocol reported by Marbán *et al*.^10^ with modifications. RA appendage tissue samples were minced and mildly digested with collagenase (Collagenase NB4 Standard Grade from *Clostridium histolyticum*, Serva Electrophoresis). Afterwards explants were generated and plated on fibronectin-coated (25 µg/mL) culture plates in cardiac explant medium (CEM) (Iscove’s modified Dulbecco’s medium (IMDM) supplemented with 20% FBS, 1% penicillin/streptomycin (5000 units/mL penicillin, 5000 µg/mL streptomycin), 2 mM L-glutamine (200 mM) and 0.1 mM 2-mercaptoethanol (50 mM)). Tissue samples obtained from young donor patients were directly minced into explants without collagenase digestion. After 2 to 3 weeks, phase-bright cells growing on top of the cardiac fibroblast outgrowth, were collected and cultured at low density (15,000–30,000 cells/mL) on poly-D-Lysin-coated (Millipore) culture plates in cardiosphere-growing medium (CGM) (35% IMDM and 65% D-MEM-F12 supplemented with 10% FBS, 1% penicillin/streptomycin (5000 units/mL penicillin, 5000 µg/mL streptomycin), 2 mM L-glutamine (200 mM), 0.1 mM 2-mercaptoethanol (50 mM) and 1X B-27 supplement (50X)). After ca. 5 days, cardiospheres were collected and plated on fibronectin-coated (25 µg/mL) culture plates in CGM medium. Phase-bright cells were harvested from individual explant cultures every 3 to 5 days up to 4 consecutive times. Medium of CDCs was changed twice a week and cells were passaged when 90% confluency was reached.

### Sequencing and data analysis

Five patient samples per group were included in the sequencing analysis (c-kit^pos^ yCPC: yCPC11, yCPC12, yCPC13, yCPC14, yCPC15; c-kit^pos^ aCPC: aCPC13, aCPC14, aCPC15, aCPC16, aCPC17; yCDC: yCDC2, yCDC3, yCDC4, yCDC6, yCDC7; aCDC: aCDC1, aCDC2, aCDC4, aCDC5, aCDC6). Total RNA was extracted using the Qiagen RNeasy micro kit with DNase treatment, followed by a clean-up step with the Qiagen RNeasy minElute columns. Quality control was performed using the Nanodrop Spectrophotometer (A260/280 > 1.8 and A260/230 > 1.5) and Agilent Bioanalyzer (RIN-value > 7). Each sample was sequenced individually on the NextSeq. 500 (High Output, 75 bp, SR; Illumina) using the Illumina TruSeq® Stranded mRNA Sample Prep Kit and 250 ng as input RNA. Reads were pre-processed accordingly and aligned with Tophat v2.0.13 to the reference genome of Homo Sapiens (GRCh37.73). The number of reads in the alignments that overlap with gene features were counted with featureCounts 1.4.6. Raw counts were further corrected within samples for GC-content and between samples using full quantile normalization, as implemented in the EDASeq package from Bioconductor. The Spearman correlation was calculated between all samples using the normalized counts as expression values.

With the EdgeR 3.8.6 package of Bioconductor, a negative binomial generalized linear model (GLM) was fitted against the normalized counts. Differential expression was calculated with a GLM likelihood ratio test in EdgeR. The resulting *P*-values were corrected for multiple testing with Benjamini-Hochberg (FDR, False Discovery Rate). Differential expression was calculated between aCDCs versus yCDCs and between aCDCs and c-kit^pos^ aCPCs. Genes with a FDR *P* < 0.05 and a fold change of |1| or more was considered significant.

String network analysis was performed on the top significant genes^37^. Clustering on the network analysis was performed using the MCL algorithm with inflation parameter set to 3. Gene ontology analysis to identify underlying biological processes was performed using DAVID Functional Annotation Tool 6.8^38^. All differentially expressed genes were submitted to Ingenuity Pathway Analysis (IPA). The top canonical pathways, significantly enriched by the differentially expressed genes, were selected by performing a Fisher Exact *t*-test (*P* < 0.05) on the ratio of enriched genes divided by the total number of genes in the pathway. A z-score statistic was calculated to predict the activation state of the pathway based on the fold change of the genes included in that pathway, with a cut-off z-score of >|1.5|. The sequencing data has been made available through the GEO website.

### Real-time qRT-PCR

Total RNA from cultured cells was isolated using RNeasy Micro Kits (Qiagen) with DNase treatment. The first strand reverse transcription was performed using GoScript Reverse Transcription System (Promega). Real-time qRT-PCR was performed using TaqMan Fast Universal PCR Master Mix and TaqMan Gene Expression Assays on a StepOnePlus System (Applied Biosystems). All reactions were run in duplicate and results were expressed as 2^−ΔΔCt^. Relative gene expression was normalized to the housekeeping genes *ACTB* and *GAPDH* and an interplate calibrator was used to compare results between plates.

### Flow cytometry analysis

CDCs and c-kit^pos^ CPCs were trypsinized at passage 5 to 7 and 50,000–100,000 cells were incubated with fluorochrome-conjugated antibodies (Table S1). Samples were evaluated on a FACSCanto II flow cytometer (BD Biosciences) with at least 10,000 recorded events. Cellular debris and aggregates were gated out based on forward and side scatter. Data were analysed with FACSDiVa software (BD Biosciences).

### *In vitro* proliferation potential

Growth kinetics were determined by calculating cell numbers at every passage to derive expansion curves and PDT throughout the entire expansion process. PDT was computed by linear regression of log2 values of cell number. Only values in the exponential growth phase were used.

### *In vitro* vasculogenic potential

Tube formation assay was performed as described previously^39^. Briefly, 65,000 c-kit^pos^ CPCs or CDCs per well were plated on 24-well plates coated with Matrigel® basement membrane matrix (BD Biosciences). Tube formation analysis was performed after 6 hours of incubation through phase-contrast mosaic images (Axiovert 200 M imaging microscope, Carl Zeiss). Angiogenesis analyser software (Gilles Carpentier) for ImageJ (National Institutes of Health) was used for morphometric analysis of the obtained images. Additionally, a 3D sprouting assay in Matrigel® was performed using spheroids as described previously^40^. Per group, cells of 2 different donors were used and for each donor, 4 different spheroids were imaged. C-kit^pos^ CPC and CDC spheroids containing 5,000 cells were formed in culture medium supplemented with poly(vinyl alcohol) in V-shaped 96-well plates for 96 hours. Formed spheroids were transferred to glass bottom culture plates coated with Matrigel®. A second layer of Matrigel® was added to accomplish a 3D structure. The sprouting was observed after 48 hours incubation through phase-contrast images (Confocal laser scanning microscope LSM 800, Carl Zeiss). To visualize the 3D sprouting, a Z-stack was made.

### Immunocytochemical analysis

For immunofluorescence staining, cells were fixed with 4% paraformaldehyde (PFA), permeabilized with 0.2% Triton-X100 and incubated with blocking solution (2% BSA) for one hour at RT. Next, cells were incubated overnight at 4 °C with primary antibodies, washed and incubated with secondary antibodies for 1 hour at RT. Secondary antibodies were conjugated with biotin to allow amplification of the signal (Table S2). For amplification, the tyramide signal amplification kit (TSA, Perkin Elmer) was used. Nuclei were stained with DAPI. Images were obtained using an Axiovert 200 M imaging microscope (Carl Zeiss).

### Paracrine assay

To compare the production of growth factors and cytokines, CDCs and c-kit^pos^ CPCs were seeded in 6-well culture plates in PBS with 2% FBS for 24 hours. Conditioned medium was collected and the concentrations of VEGF, bFGF, IGF-1, HGF, PlGF and SDF-1 were measured using human-specific ELISA kits (R&D Systems), according to the manufacturer’s instructions. In addition, total protein levels were determined by a bicinchoninic acid assay (Pierce BCA Protein Assay Kit, Thermo Scientific), according to the manufacturer’s instructions to normalize the production of growth factors and cytokines.

### Conditioned medium and exosome purification

CDCs (yCDC, n = 2; aCDC, n = 2) and c-kit^pos^ CPCs (c-kit^pos^ yCPC, n = 3; c-kit^pos^ aCPC, n = 3) were cultured for 48 hours in serum-free medium, before collection conditioned medium. Exosomes were isolated using the ultracentrifugation method. Briefly, cells were pelleted by low speed centrifugation (15 min. at 500 g). The supernatant, containing the exosomes and cell debris, were then centrifuged for 30 min. at 10,000 g to pellet the cell debris. The residual supernatant was then finally ultracentrifuged in 2 different steps (3 hours at 140,000 g, 70 min. at 140,000 g) to obtain the exosome fraction. The purified exosome pellet was dissolved in PBS for further analysis. All centrifugation and ultracentrifugation steps were performed at 4 °C. The nanoparticle analysis technology (Nanosight) was used to measure particle size and concentration. Six technical replicates per exosome sample were performed. The extraction of total RNA of the exosomes was performed using the total exosome RNA and protein isolation kit (Life technologies). The total exosome RNA yield and quality was measured by a spectrophotometer (NanoDrop2000, Thermo scientific). Normalized expression levels of miR-22-3p, miR-132-3p, miR-146a-3p and miR-210-3p were calculated using miR-16 as a housekeeping gene.

### *In vitro* differentiation assays of mono-culture of human stem cells

To induce CM differentiation, c-kit^pos^ CPCs and CDCs were seeded at densities of 5 × 10^3^ cells/cm^2^. The c-kit expansion or CGM maintenance medium (CON) was replaced by cardiomyocyte differentiation medium (Millipore) (DIFF) for 12 days with fractional medium changes of 50% every 2–3 days. Cells were stained for sarcomeric alpha-actinin and F-actin to determine CM differentiation. Images were obtained using an Axiovert 200 M imaging microscope (Carl Zeiss) and quantification was done using ImageJ software (National Institutes of Health).

### Culture conditions SWAP experiment

c-kit^pos^ CPCs were cultured in CDC expansion medium (35% IMDM and 65% D-MEM-F12 supplemented with 10% FBS, 1% penicillin/streptomycin (5000 units/mL penicillin, 5000 µg/mL streptomycin), 2 mM L-glutamine (200 mM), 0.1 mM 2-mercaptoethanol (50 mM) and 1X B-27 supplement (50X)) and fibronectin-coated (25 μg/mL) culture plates and CDCs in c-kit^pos^ CPC expansion medium (Ham’s F12 medium supplemented with 10% FBS, 1% penicillin/streptomycin (5000 units/mL penicillin, 5000 µg/mL streptomycin), 5 mU/mL human erythropoietin (EPO)) for at least 2 passages. Total RNA from cultured cells was isolated and qRT-PCR was performed. In addition, cells were plated on Matrigel® basement membrane matrix-coated culture plates for a tube formation assay.

### Statistical analysis

All data are presented as mean ± standard error of the mean (SEM) except for the RT-qPCR results, which are presented as geometric mean with 95% confidence interval (CI). Intergroup differences were analysed using a two-tailed unpaired t-tests or one-way ANOVA followed by a Bonferroni post-hoc test or a chi-square test for normally distributed data. Non-normally distributed data were compared using a non-parametric Mann-Whitney test or non-parametric Kruskal-Wallis test followed by a Dunn’s post-hoc test. Normality of the data was tested using a Shapiro-Wilk normality test. Spearman non-parametric correlation was used to analyse linear co-variation. The goodness of fit was expressed as the coefficient of determination R^2^. A probability value of *P* < 0.05 was considered statistically significant. All analyses were performed using Prism 6.0e software (GraphPad Software).

### Data availability

The sequencing data generated and analysed during the current study has been made available through the GEO website.

## Electronic supplementary material

Supplementary Information
