# Supplementary material for: Molecular signature of progenitor cells isolated from young and adult human hearts
Source: Sci Rep. 2018 Jun 18;8:9266. doi: 10.1038/s41598-018-26969-2 (PMC6006291; doi:10.1038/s41598-018-26969-2)
Supplement: Supplementary file 1 — Supplementary Information [file 41598_2018_26969_MOESM1_ESM.pdf]

Molecular signature of progenitor cells isolated from young and adult human hearts

Ann-Sophie Walravens[1], Maarten Vanhaverbeke[1], Lara Ottaviani[1], Hilde Gillijns[1], Sander Trenson[1], Nina Vanden Driessche[1], Aernout Luttun[1], Bart Meyns, Paul Herijgers, Filip Rega, Ruth Heying, Maurilio Sampaolesi[2], and Stefan Janssens*[1]

Supplemental data information

Figure S1

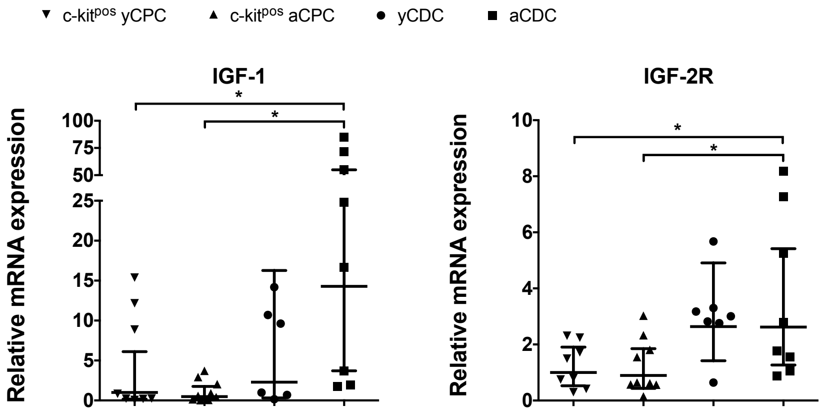

Figure S1. Gene expression levels of *IGF-1* and *IGF-2R* of young and adult c-kit^{pos} CPCs and CDCs
c-kit^{pos} yCPC (n=8), c-kit^{pos} aCPC (n=9), yCDC (n=7) and aCDC (n=7). Results are geometric mean with 95% CI, Kruskal-Wallis test for non-normally distributed data, *p<0.05. CPC, cardiac progenitor cell; CDC, cardiosphere-derived cell.

Figure S2

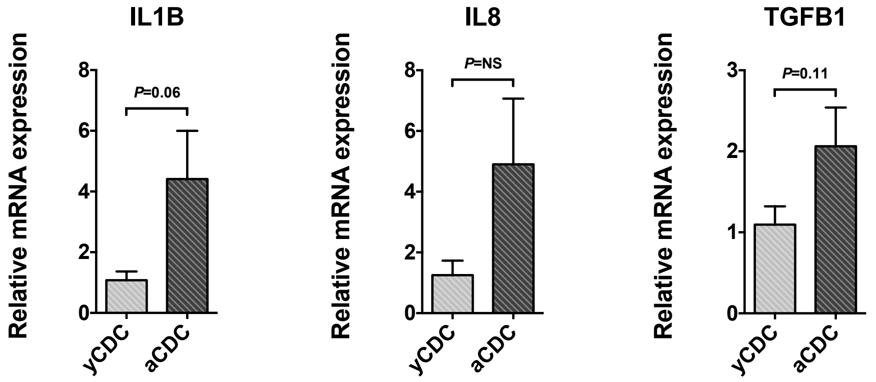

Figure S2. Gene expression confirmation of *IL1B, IL8* and *TGFB1* of adult c-kit^{pos} CPCs and young and adult CDCs
Confirmation of gene expression of *IL1B, IL8* and *TGFB1* comparing yCDC (n=4) and aCDC (n=4). Results are shown as mean±SEM. CDC, cardiosphere-derived cell.

Figure S3

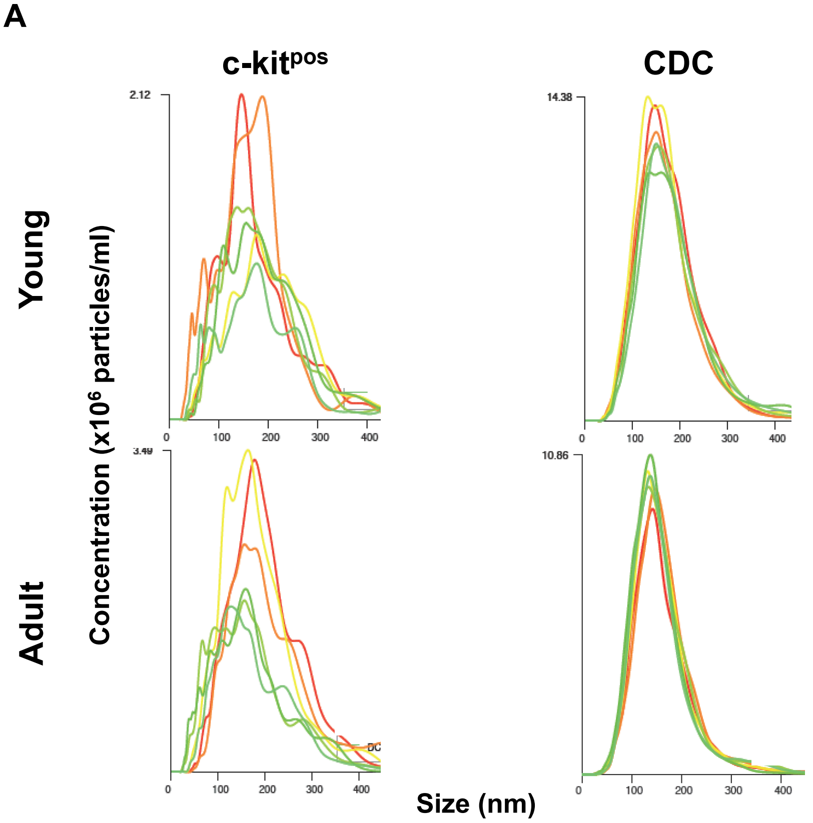
A
c-kit^pos
CDC
Young
Adult
Concentration (x10^6 particles/ml)
Size (nm)
2.12
14.38
3.49
10.86
0
100
200
300
400

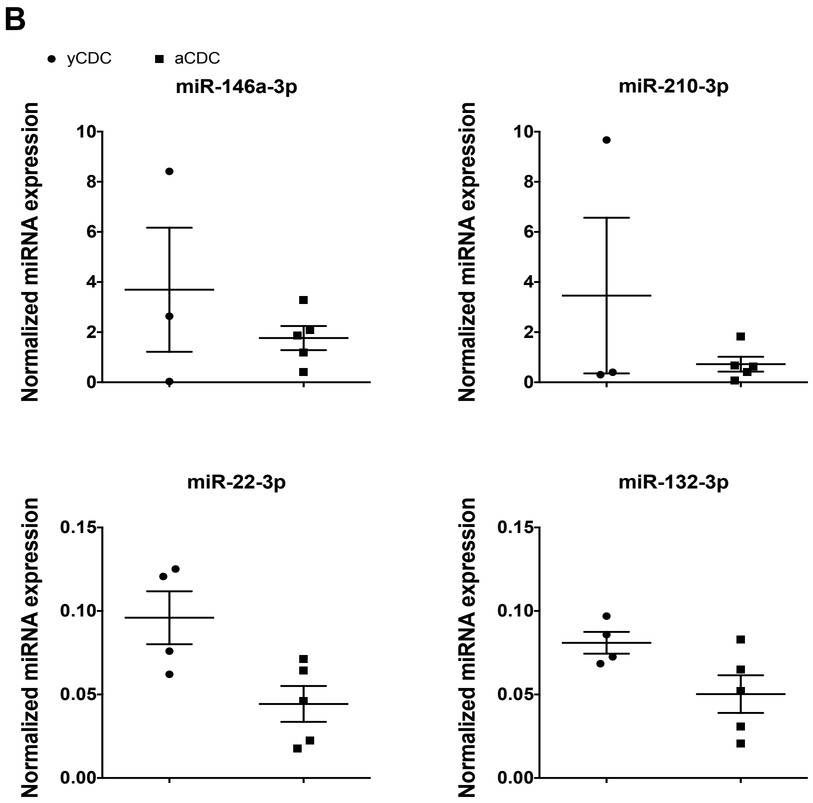
B
yCDC
aCDC
miR-146a-3p
miR-210-3p
miR-22-3p
miR-132-3p
Normalized miRNA expression
0
2
4
6
8
10
0.00
0.05
0.10
0.15

Figure S3. Characterization of exosomes derived from adult and young c-kit^{pos} CPCs and CDCs.

(**A**) Nanoparticle analysis of c-kit^{pos} CPCs (c-kit^{pos} yCPC, n=3; c-kit^{pos} aCPC, n=3) and CDCs (yCDC, n=2; aCDC, n=2)-derived nanoparticles to determine size distribution and concentration. Depicted is the nanoparticle analysis of a representative exosome sample of each group. Each image shows 6 technical replicates of that sample. CPC, cardiac progenitor cell; CDC, cardiosphere-derived cell. (**B**) Normalized expression levels of miR-22-3p, miR-132-3p, miR-146a-3p and miR-210-3p present in young (n=4) and adult (n=5) CDC-derived exosomes. CDC, cardiosphere-derived cell.

Figure S4

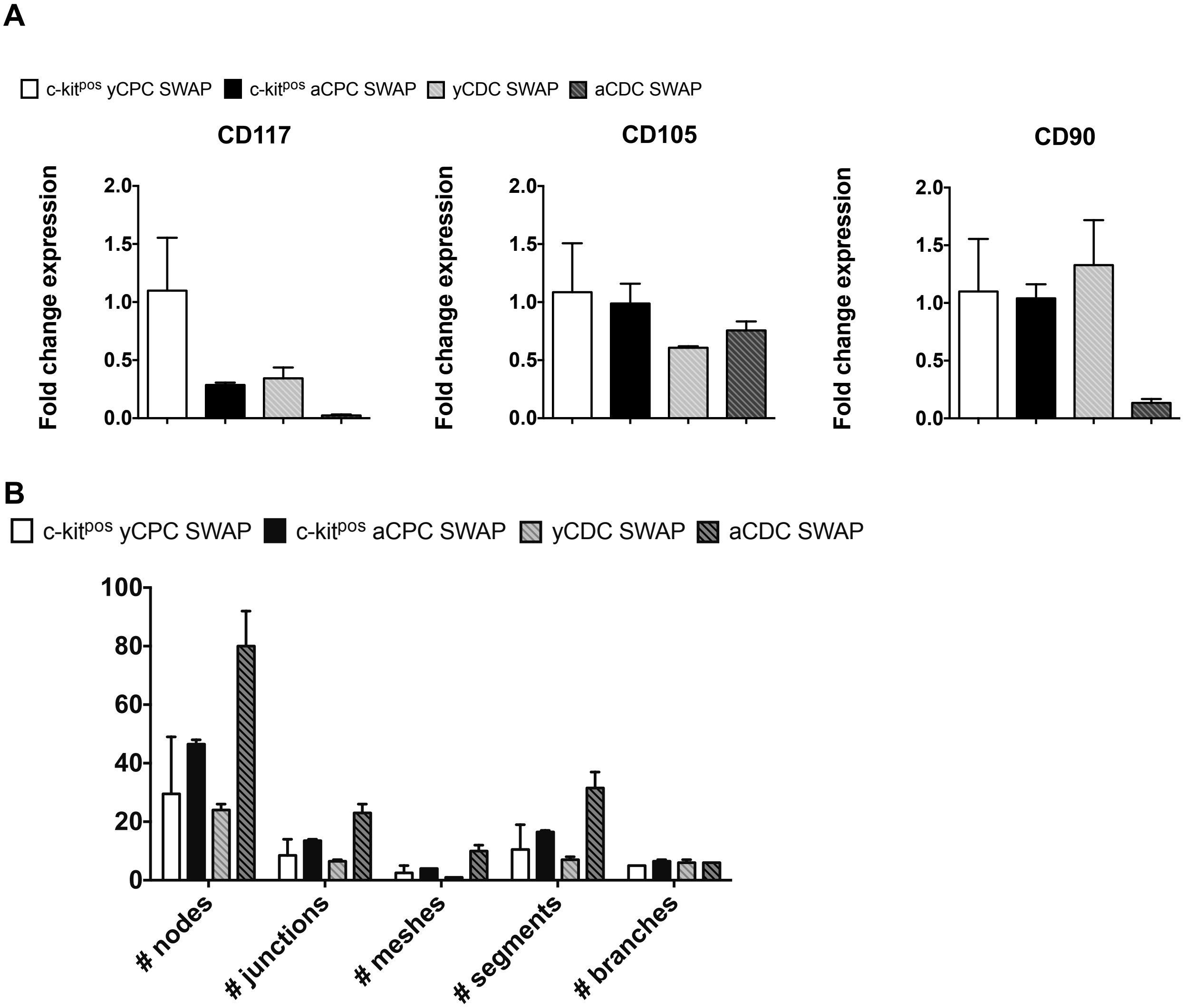

Figure S4. Phenotypical and functional differences between c-kit^{pos} CPCs and CDCs are independent from culture conditions.

(**A**) Normalized expression levels of *CD117*, *CD105* and *CD90* in c-kit^{pos} CPCs (young, n=2; adult, n=2) and CDCs (young, n=2; adult, n=2) after culture in their counterpart expansion medium for at least 2 passages. Results are mean±SEM. CPC, cardiac progenitor cell; CDC, cardiosphere-derived cell. (**B**) Analysis of network formation 6 hours after culture on Matrigel® of c-kit^{pos} yCPCs (n=2), c-kit^{pos} aCPCs (n=2), yCDCs (n=2) and aCDCs (n=2). Quantification of number of nodes, junctions, meshes, segments and branches normalized to analysed area. Results are mean±SEM. SWAP, CDCs cultured in c-kit^{pos} expansion medium and c-kit^{pos} CPCs

cultured in CDC expansion medium and fibronectin-coated culture plates; CPC, cardiac progenitor cell; CDC, cardiosphere-derived cell.

Table S1. Overview of monoclonal antibodies used for flow cytometry

Monoclonal antibody	Fluorochrome	Incubation Temp.	Supplier (catalog N°)
CD117 (1:100)	APC	4°C	DAKO (C7244)
CD105 (1:100)	APC	4°C	BD Pharmingen (562404)
CD90 (1:200)	APC	RT	BD Pharmingen (559869)

APC indicates allophycocyanin.

Table S2. Overview of primary antibodies, secondary antibodies and amplification methods used for immunofluorescence stainings of cells

Target	Primary Antibody	Secondary Antibody	Amplification
α-sarcomeric actinin	Abcam (1:100)	RAM-Biotin (1:200)	TSA
F-actin (phalloidin)	Molecular Probes (1:50)	-	-

RAM indicates rabbit anti-mouse; TSA, tyramide signal amplification.

Table S3. Detailed clinical characteristics of the patients

Patient	Age	Gender	Cardiovascular risk factors				Aetiology
			Hypertension	Diabetic	Hyperlipidaemia	Smoking	
yCPC1	2,5 y	M	N/A	N/A	N/A	N/A	ASD
yCPC2	14 y	F	N/A	N/A	N/A	N/A	ASD
yCPC3	9 m	F	N/A	N/A	N/A	N/A	VSD
yCPC4	7 m	F	N/A	N/A	N/A	N/A	AVSD
yCPC5	8,5 m	M	N/A	N/A	N/A	N/A	AVSD
yCPC6	5,5 m	M	N/A	N/A	N/A	N/A	VSD
yCPC7	6 d	F	N/A	N/A	N/A	N/A	VSD
yCPC8	4 m	F	N/A	N/A	N/A	N/A	Tetralogy of Fallot
yCPC9	3 d	F	N/A	N/A	N/A	N/A	TGA
yCPC10	3 m	F	N/A	N/A	N/A	N/A	VSD
yCPC11	2 m	F	N/A	N/A	N/A	N/A	VSD
yCPC12	5 d	F	N/A	N/A	N/A	N/A	TGA
yCPC13	3 m	F	N/A	N/A	N/A	N/A	Tetralogy of Fallot
yCPC14	2.5 m	F	N/A	N/A	N/A	N/A	Tetralogy of Fallot
yCPC15	4 m	M	N/A	N/A	N/A	N/A	AVSD
aCPC1	80 y	F	Yes	Pre-diabetic	Yes	No	MVP/MVR ± CABG
aCPC2	70 y	M	Yes	No	Yes	Ex-smoker	MVP/MVR ± CABG
aCPC3	53 y	M	Yes	Pre-diabetic	Yes	Ex-smoker	MVP/MVR ± CABG
aCPC4	57 y	M	Yes	No	Yes	Ex-smoker	Other
aCPC5	81 y	M	Yes	No	Yes	No	Complex valvular disease
aCPC6	73 y	M	Yes	Diabetic	No	Active	MVP/MVR ± CABG
aCPC7	73 y	M	Yes	Diabetic	Yes	No	AVP/AVR ± CABG

**aCPC8**	68 y	M	Yes	Diabetic	Yes	Ex-smoker	Complex valvular disease
**aCPC9**	75 y	F	Yes	Pre-diabetic	Yes	No	Complex valvular disease
**aCPC10**	80 y	M	No	No	Yes	No	AVP/AVR ± CABG
**aCPC11**	84 y	F	Yes	No	Yes	No	Complex valvular disease
**aCPC12**	79 y	M	Yes	No	Yes	Ex-smoker	AVP/AVR ± CABG
**aCPC13**	74 y	F	Yes	Yes	Yes	No	Complex valvular disease
**aCPC14**	62 y	F	Yes	No	Yes	Ex-smoker	MVP/MVR ± CABG
**aCPC15**	65 y	F	Yes	Yes	Yes	Yes	AVP/AVR ± CABG
**aCPC16**	82 y	F	Yes	No	Yes	No	Complex valvular disease
**aCPC17**	79 y	F	Yes	No	No	No	MVP/MVR ± CABG
**aCDC1**	70 y	M	Yes	Pre-diabetic	Yes	No	MVP/MVR ± CABG
**aCDC2**	47 y	M	No	Unknown	Yes	Active	AVP/AVR ± CABG
**aCDC3**	61 y	F	No	No	Yes	No	MVP/MVR ± CABG
**aCDC4**	77 y	M	Yes	No	No	Active	Complex valvular disease
**aCDC5**	75 y	F	Yes	No	Yes	No	Complex valvular disease
**aCDC6**	65 y	M	Yes	Pre-diabetic	No	Ex-smoker	MVP/MVR ± CABG
**aCDC7**	56 y	F	No	No	Yes	No	MVP/MVR ± CABG
**aCDC8**	49 y	M	No	No	No	No	AVP/AVR ± CABG
**aCDC9**	60 y	M	Yes	No	Yes	No	Complex valvular disease
**yCDC1**	11 m	M	N/A	N/A	N/A	N/A	AVSD
**yCDC2**	4 d	M	N/A	N/A	N/A	N/A	TGA
**yCDC3**	5 m	M	N/A	N/A	N/A	N/A	Tetralogy of Fallot
**yCDC4**	10 y	M	N/A	N/A	N/A	N/A	ASD
**yCDC5**	4 m	F	N/A	N/A	N/A	N/A	Tetralogy of Fallot
**yCDC6**	1,5 m	F	N/A	N/A	N/A	N/A	Tetralogy of Fallot
**yCDC7**	3 m	M	N/A	N/A	N/A	N/A	Tetralogy of Fallot

CPC indicates cardiac progenitor cell; CDC, cardiosphere-derived cell; y, years; m, months; d, days; M, male; F, female; ASD, atrial septal defect; VSD, ventricular septal defect; AVSD, atrioventricular septal defect; TGA, transposition of the great arteries, MVP/R, mitral valve repair/replacement; AVP/R, aortic valve repair/replacement; CABG, coronary artery bypass grafting; N/A, Not Applicable
